# Intravenous Ferric Chloride Hexahydrate Supplementation Induced Endothelial Dysfunction and Increased Cardiovascular Risk among Hemodialysis Patients

**DOI:** 10.1371/journal.pone.0050295

**Published:** 2012-12-05

**Authors:** Ko-Lin Kuo, Szu-Chun Hung, Yao-Ping Lin, Ching-Fang Tang, Tzong-Shyuan Lee, Chih-Pei Lin, Der-Cherng Tarng

**Affiliations:** 1 Department and Institute of Physiology, National Yang-Ming University, Taipei, Taiwan; 2 Institute of Clinical Medicine, National Yang-Ming University, Taipei, Taiwan; 3 Department of Biotechnology and Laboratory Science in Medicine and Institute of Biotechnology in Medicine, National Yang-Ming University, Taipei, Taiwan; 4 School of Medicine, Tzu Chi University, Hualien, Taiwan; 5 Division of Nephrology, Buddhist Tzu Chi General Hospital, Taipei Branch, Taipei, Taiwan; 6 Division of General Laboratory, Department of Pathology and Laboratory Medicine, Taipei Veterans General Hospital, Taipei, Taiwan; 7 Division of Nephrology, Department of Medicine and Immunology Centre, Taipei Veterans General Hospital, Taipei, Taiwan; Integrated Research Centre, Germany

## Abstract

**Background:**

The association between intravenous (IV) iron administration and outcomes in hemodialysis (HD) patients is still debated. Therefore, this study was aimed to assess the relationship between the IV administration of ferric chloride hexahydrate (Atofen®) and cardiovascular (CV) outcome and the interaction between iron-induced oxidative stress and endothelial dysfunction in chronic HD patients.

**Methodology/Principal Findings:**

A cohort of 1239 chronic HD patients was recruited. In a follow-up of 12 months, Kaplan-Meier survival curves showed that higher doses of IV Atofen associated with higher risks for CV events and deaths in HD patients. In multivariate Cox models, compared to no iron supplementation, IV Atofen administration was an independent predictor for CV events and overall mortality. However, the nature of the observational cohort study possibly bears selection bias. We further found that IV Atofen enhanced the superoxide production of mononuclear cells (MNCs), the levels of circulating soluble adhesion molecules, and the adhesion of MNCs to human aortic endothelial cells (HAECs). *In vitro* experiments showed that Atofen increased the expression of intracellular cell adhesion molecule-1 and vascular cell adhesion molecule-1 in HAECs and aggravated the endothelial adhesiveness in a dose-dependent manner. These iron-induced changes were significantly attenuated by the co-treatment of HAECs with N-acetylcysteine and inhibitors of NADPH oxidase, nuclear factor κB, and activator protein-1.

**Conclusion:**

A cumulative dose of IV Atofen >800 mg within 6 months was associated with an adverse CV outcome and a higher mortality among chronic HD patients. The detrimental effects of IV iron supplementation were partly due to the increased oxidative stress and induction of MNC adhesion to endothelial cells, a pivotal index of early atherogenesis.

## Introduction

Anemia is associated with cardiovascular (CV) and global outcomes in patients with chronic kidney disease (CKD) [1]. The correction of anemia requires erythropoiesis-stimulating agents (ESAs) in most instances. The potential role of intravenous (IV) iron therapy in enhancing the efficacy of ESAs in CKD patients has received increasing attention in recent years [2]. IV iron therapy is valuable because it helps to reduce ESA requirements, allows dialysis patients to achieve increased hemoglobin levels, and increases the cost-effectiveness of ESA treatment [3], [4]. However, iron is a transition metal and potent pro-oxidant that is capable of redox cycling. Acute high IV iron dose administration may provoke the generation of bioactive iron (non-transferrin-binding iron), subsequently elevate reactive oxygen species (ROS) in plasma and reduce the forearm flow-mediated dilatation in healthy individuals [5]. Drueke *et al*. [6] disclosed an interrelation of the annual IV iron dose administered with the common carotid artery intima-media thickness and with the generation of advanced oxidation products from proteins in hemodialysis (HD) patients. It is speculated that the chronic cumulative administration of IV iron might exaggerate oxidative stress and potentially increase the CV risk in CKD patients.

**Table 1:** Baseline characteristics of hemodialysis patients after receiving 6-month of IV administration of ferric chloride hexahydrate or not.

Parameters	Non-IV Iron group (n = 583)	IV Iron group (n = 656)	*P* value
Age, years	58±14	59±14	0.078^a^
Male sex, n (%)	271 (46)	308 (47)	0.830^b^
Diabetes mellitus, n (%)	167 (29)	165 (25)	0.176^b^
Hypertension, n (%)	244 (42)	256 (39)	0.332^b^
Prior cardiovascular disease, n (%)	87 (15)	93 (14)	0.667^b^
Causes for chronic renal failure			
Glomerulonephritis, n (%)	280 (48)	302 (46)	0.636^b^
Interstitial nephritis, n (%)	47 (8)	66 (10)	0.361^b^
Diabetic nephropathy, n (%)	157 (27)	164 (25)	0.511^b^
Nephrosclerosis, n (%)	41 (7)	39 (6)	0.336^b^
Others, n (%)	58 (10)	85 (13)	0.419^b^
Statin, n (%)	98 (17)	123 (19)	0.473^b^
ACEI and/or ARB, n (%)	232 (40)	250 (38)	0.744^b^
Other antihypertensives, n (%)	167 (27)	183 (28)	0.608^b^
Hemodialysis vintage, months	56±44	60±50	0.134^a^
Albumin, g/dL	4.0±0.4	4.0±0.40	0.189^a^
Total cholesterol, mg/dL	182±48	182±44	0.888^a^
Triglyceride, mg/dL	171±131	163±117	0.259^a^
Fasting glucose, mg/dL	139±74	131±68	0.063^a^
C-reactive protein, mg/L	5.21 [0.40, 9.82]	4.8 [0.30, 9.65]	0.103^c^
Hemoglobin, g/dL	10.4±1.5	10.2±1.7	0.062^a^
Erythropoietin dose, U/kg/week	70.3±41.1	70.6±34.9	0.888^a^
IV Iron dose, mg/6months	0 [0, 0]	320 [160, 1000]	
0 mg, n (%)	583 (100)	0 (0)	
40 to 800 mg, n (%)		451 (67)	
840 to 1600 mg, n (%)		111 (20)	
1640 to 2400 mg, n (%)		94 (13)	

Abbreviations: ACEI, angiotensin converting enzyme inhibitor; ARB, angiotensin II receptor blockade; IV, intravenous; nPCR, normalized protein catabolic rate; PTH, parathyroid hormone.

Other antihypertensives consisted of calcium channel blockers, β blockers, α blockers and diuretics.

Values of C-reactive protein, and IV iron dose are reported as medians with interquartile ranges; other continuous variables are reported as means ± SD.

Comparisons between two groups by ^a^Student’s t test;

b Person’s chi-square test, and ^c^Mann-Whitney U test.

So far, there is no definitive safe upper limit of the cumulative dose of IV iron. Three observational studies in large study cohorts did not provide definitive conclusions. Feldman *et al*. first showed a significantly increased mortality in 5833 HD patients receiving an IV iron dose >1000 mg over a 6-month period [7]. In contrast, in a different cohort of 32,566 HD patients, Feldman *et al*. failed to identify a statistically significant association between iron administration and mortality [8]. Kalantar-Zadeh *et al.* analyzed data from a cohort of 58,038 HD patients and found, after appropriate adjustments, that patients receiving a monthly IV iron dose >400 mg had significantly increased global and CV deaths compared with those who received no IV iron [9]. Accordingly, the adverse effects of iron supplementation on CV outcomes in CKD patients remain a matter of debate. Today, evidence from experimental studies to support the toxicity of iron to cells or tissues is still fragmentary and contradictory.

**Figure 1:**
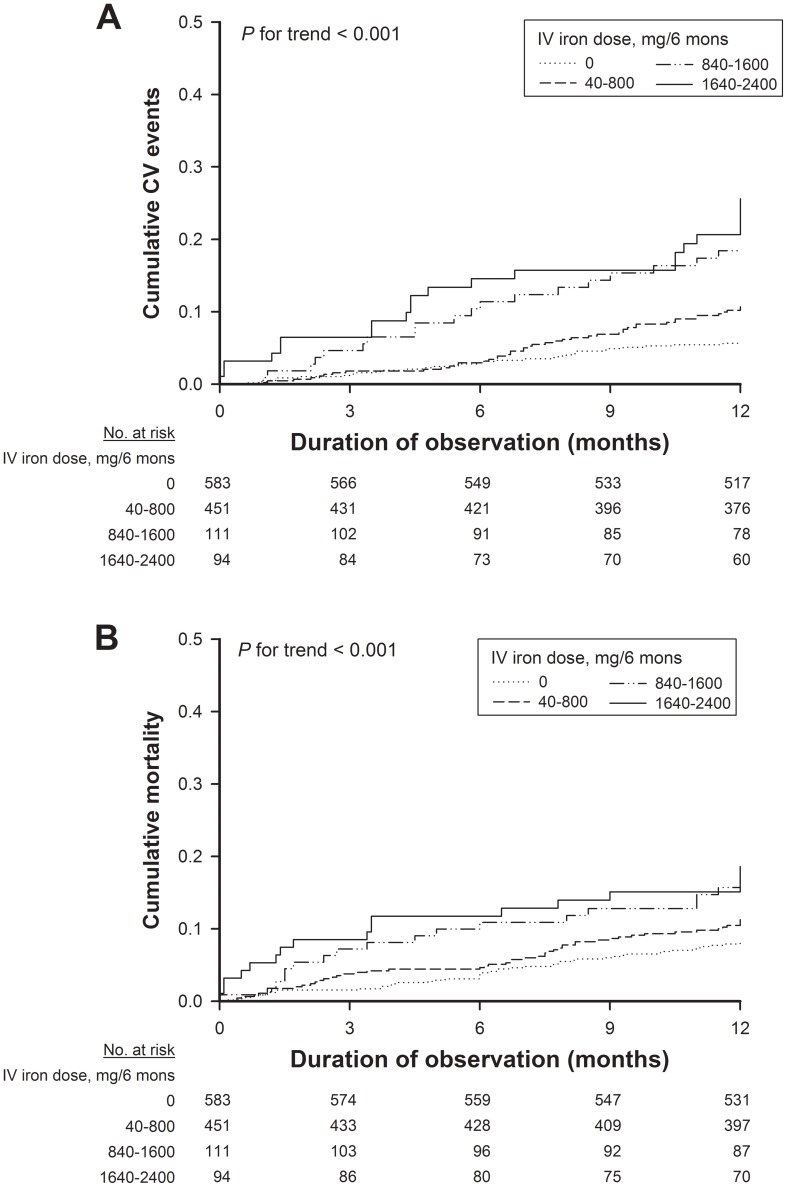
IV iron was associated with CV events and overall mortality. Kaplan-Meier curves demonstrated cumulative CV events (A) and overall mortality (B) among 1239 hemodialysis patients. All patients were stratified into 4 groups according to the cumulative dose of IV iron within the 6 months prior to the enrollment.

In the early stage of atherosclerosis, adhesion molecules, such as vascular cell adhesion molecule-1 (VCAM-1), intracellular cell adhesion molecule-1 (ICAM-1), and endothelial cell selectin (E-selectin), are expressed on the endothelial cell surface to facilitate the adhesion of circulating mononuclear cells (MNCs) to endothelial cells and their subsequent migration to the subendothelial space. ROS serve as common intracellular messengers for the redox-sensitive pathways and induce the expression of adhesion molecules in vascular endothelial cells [10], [11]. Iron provokes ROS production *in vivo* [6], [12]. Therefore, it seems rational to postulate that IV iron can promote early atherogenesis by increasing the expression of adhesion molecules and the adhesion of MNCs to endothelial cells. This speculation deserves further clinical studies as well as *in vitro* experiments.

**Table 2:** Multivariate Cox proportional hazards analysis for relative risk of cardiovascular events and overall mortality calculated for intravenous iron groups in a follow-up of 12 months.

	Intravenous iron administered dose, mg/6 months				
	0	40 to 800	840 to 1600	1640 to 2400	
	Hazard Ratio (95% CI)				*P* for trend
**Cardiovascular events**					
Crude	1	1.8 (1.1–2.9)	3.4 (1.9–6.0)	4.8 (2.9–8.4)	<0.001
Adjusted^a^	1	1.7 (1.0–2.7)	3.5 (1.9–6.1)	5.1 (3.0–9.7)	<0.001
**Fatal and nonfatal MI and stroke, and** **sudden death**					
Crude	1	1.4 (0.8–1.8)	1.9 (1.1–3.2)	2.5 (2.0–6.3)	0.009
Adjusted^a^	1	1.2 (0.9–1.9)	2.4 (1.2–4.7)	3.0 (1.5–5.6)	<0.001
**All-cause mortality**					
Crude	1	1.3 (0.9–2.0)	2.0 (1.1–3.4)	2.4 (1.3–4.1)	0.004
Adjusted^a^	1	1.3 (0.9–2.0)	3.1 (1.6–6.3)	3.7 (1.8–7.7)	<0.001

Hazard ratios and 95% confidence intervals (CIs) were derived from Cox regression analysis with intravenous iron dose taken into account as a time-dependent covariate.

a The multivariate model included variables for age, sex, diabetes, hypertension, prior cardiovascular disease, hemodialysis duration, serum albumin, hemoglobin, and C-reactive protein.

Therefore, first, we conducted a prospective observational study to validate the association of the cumulative 6-month dose of IV ferric chloride hexahydrate (Atofen®; Uji pharmaceutical Co. Ltd., Japan) with CV events or overall mortality among 1239 patients receiving chronic HD in a follow-up period of 12 months. Second, another HD cohort was recruited to assess the *in vivo* pro-oxidant effects of IV Atofen by measuring the intracellular production of ROS in circulating MNCs and soluble adhesion molecule levels in plasma, as well as the *ex vivo* cell adhesion between cultured human aortic endothelial cells (HAECs) and circulating MNCs following IV Atofen therapy. Finally, we carried out *in vitro* experiments to assess the effects of iron on early atherogenesis by examining the expression of ICAM-1 and VCAM-1 in HAECs treated with Atofen and the subsequent endothelial cell adhesiveness to circulating MNCs. Our study suggests a novel role of IV-administered Atofen in initiating atherosclerosis and affecting CV outcome in HD patients.

**Figure 2:**
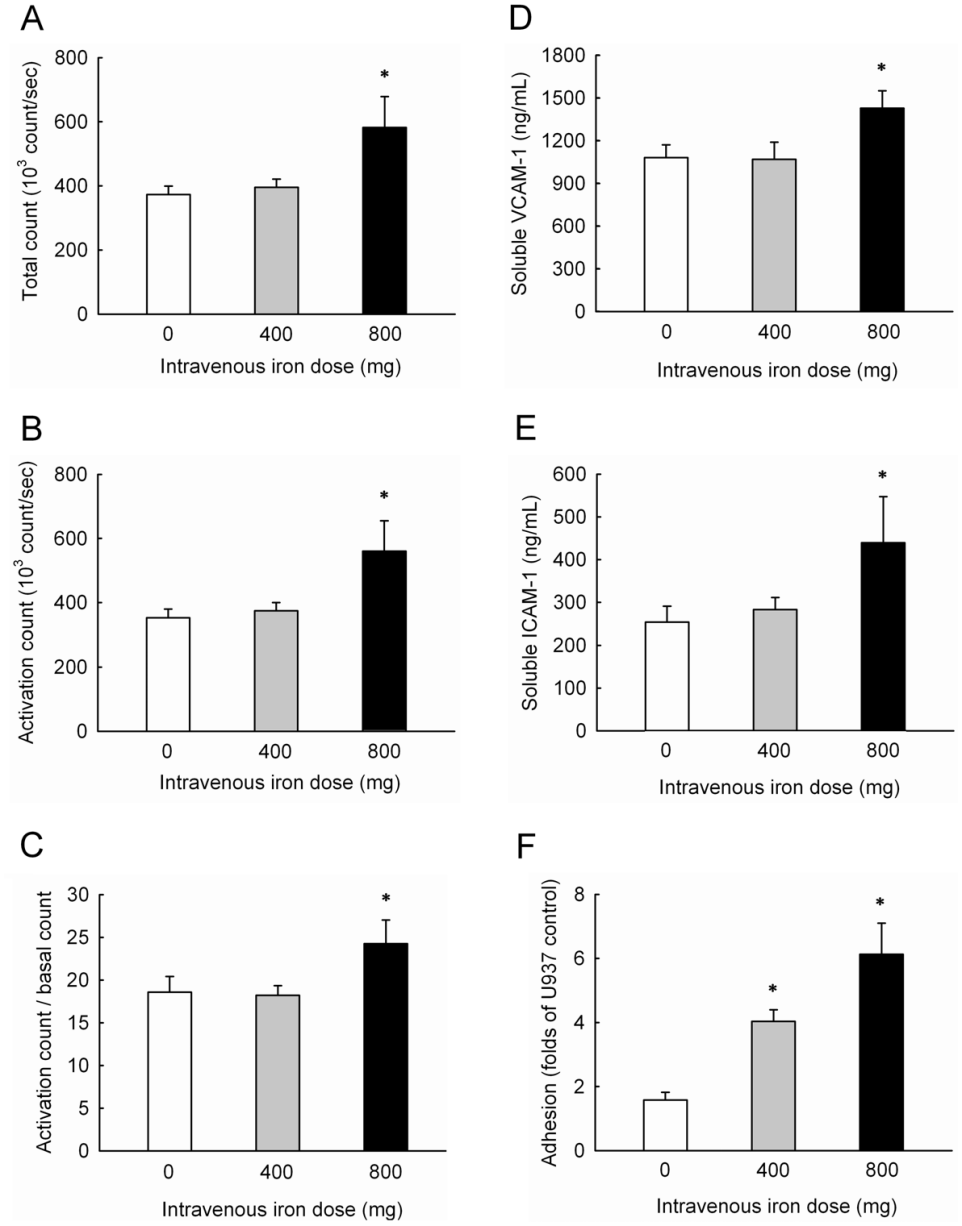
IV iron significantly increased superoxide production and endothelial damage *in vivo*. Total (A) and activated (B) intracellular production of superoxide, the ratio of activated to total superoxide (C) in circulating MNCs, plasma levels of VCAM-1 (D) and ICAM-1 (E), and endothelial adhesiveness of circulating MNCs (F) in hemodialysis patients. All patients were randomly allocated into 3 groups according to the IV Atofen iron doses of 0 mg (n  =  20), 400 mg (n  =  20), and 800 mg (n  =  20). *P<0.05 compared with patients not receiving IV iron therapy. Abbreviations: ICAM-1, intracellular cell adhesion molecule-1; VCAM-1, vascular cell adhesion molecule-1.

## Materials and Methods

### Patients and Study Protocol

This prospective cohort study was carried out at the dialysis facility of Excelsior Renal Service Co., Ltd., Taiwan, to assess the safety of IV Atofen iron supplementation in terms of CV outcome and all-cause mortality among chronic HD patients. The study subjects were recruited between January 1 and June 30, 2004. Initially, 1911 patients undergoing chronic HD were screened, and 1687 clinically stable patients older than 20 years of age with a HD vintage of more than 6 months before the study and with a life expectancy of more than 6 months were included. The medical history of each patient was reviewed and confirmed by checking patient records; one physician (H. S.-C.) did this to minimize interobserver variations. The exclusion criteria were: dialysis for less than 12 h per week; inadequacy of dialysis, defined as a Kt/V urea <1.2; malignancy, infectious disease, sepsis, hepatobiliary disease, or hospitalization within 3 months before study; and those who were unable to give informed consent. Ultimately, our study population was comprised of 1239 patients (579 men and 660 women; mean age of 59±14 years). The baseline demographic, comorbidity, and laboratory measurements were recorded at enrollment. The study subjects were followed up from July 1, 2004 to June 30, 2005. All of the patients were subjected to a standard bicarbonate dialysis session. HD was performed three times weekly using single-use dialyzers with a membrane surface area of 1.6–1.7 m^2^. Dialysis machines were sterilized daily, and water treatment circuits and tanks were sterilized weekly.

**Figure 3:**
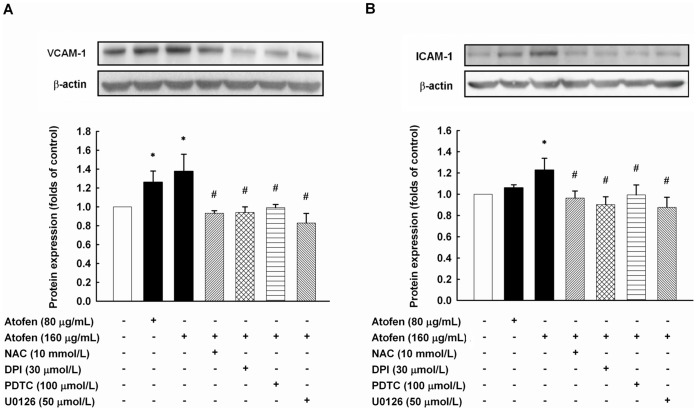
Iron upregulated the expressions of VCAM-1 and ICAM-1 in HAECs. The expressions of VCAM-1 (A) and ICAM-1 (B) in iron-stimulated HAECs were determined by Western blotting. HAECs were treated with ferric chloride hexahydrate (Atofen) with or without NAC (100 mmol/L), DPI (30 µmol/L), PDTC (100 µmol/L), and U0126 (50 µmol/L) for 4 hours. Data (mean ± SEM) are shown as a bar graph of densitometry data from 3 separate experiments. *P<0.05 compared with baseline without any treatment. ^#^ *P*<0.05 compared with 160 µg/mL ferric chloride hexahydrate for 4 h. Abbreviations: DPI, diphenyleneiodonium; HAECs, human aortic endothelial cells; ICAM-1, intracellular cell adhesion molecule-1; NAC, N-acetylcysteine; PDTC, pyrrolidine dithiocarbamate; VCAM-1, vascular cell adhesion molecule-1.

All patients were divided into two groups: with (*n*  =  656) and without (*n*  =  583) IV administration of Atofen 6 months prior to enrollment. Iron-treated patients were further stratified into 3 subgroups according to the cumulative 6-month iron dose: 40 to 800 mg (Group I, *n*  =  451), 840 to 1600 mg (Group II, *n*  =  111) and 1640 to 2400 mg (Group III, *n*  =  94), respectively. The group without IV iron treatment was defined as the reference. In a follow-up of 12 months, fatal and non-fatal CV events and all-cause deaths were recorded. In all patients, a thorough medical history was taken at the time of study enrollment. The presence of CV disease was defined with a medical history and clinical findings of congestive heart failure (CHF), coronary artery disease, or cerebrovascular and/or peripheral vascular disease (PVD). Diabetes mellitus was diagnosed according to the American Diabetes Association criteria or if the patients were taking oral hypoglycemic agents or receiving insulin injection therapy at the time of enrollment. No major modifications were made in the dialysis treatments or schedules during the follow-up period. The primary outcome measures were CV events and all-cause mortality from the time of inclusion in the study. CV events included myocardial infarction, stroke, CHF, complicated PVD, and sudden death. CHF was ascertained by the dyspnea plus two of the followings: raised jugular venous pressure, bibasilar crackles, pulmonary venous hypertension or interstitial edema on chest X-ray. Complicated PVD is defined if patients with PVD requires the hospital-based interventions, including non-traumatic amputation, percutaneous transluminal angioplasty or bypass surgery. All clinical events were confirmed by hospital records.

**Figure 4:**
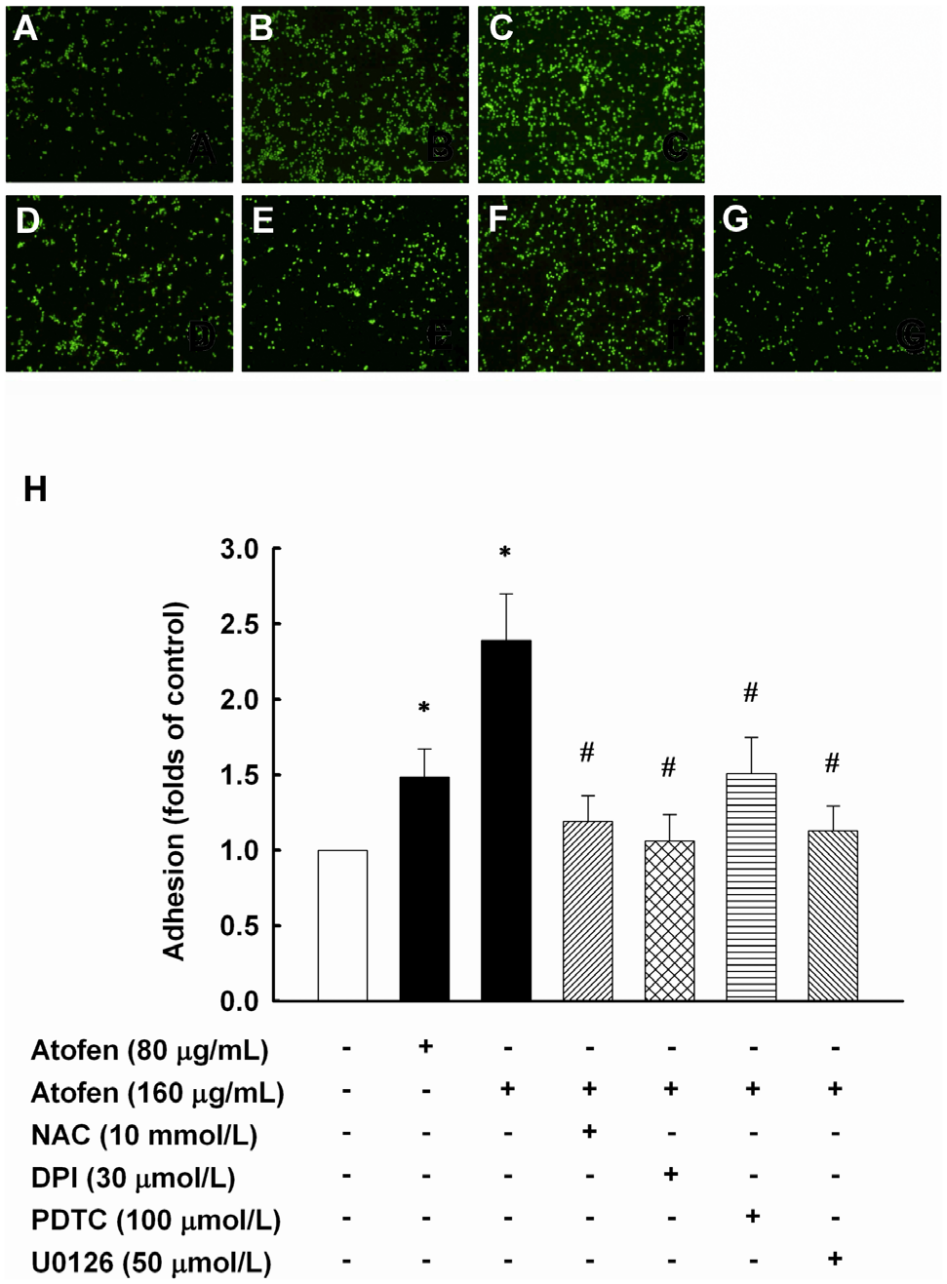
Iron enhanced adhesiveness of human MNCs to HAECs. HAECs were treated with ferric chloride hexahydrate (Atofen) with or without NAC (100 mmol/L), DPI (30 µmol/L), PDTC (100 µmol/L), and U0126 (50 µmol/L) for 4 hours. In upper panel, HAECs were unstimulated (control) (A), or stimulated by 80 µg/mL of Atofen alone (B), by 160 µg/mL of Atofen alone (C), and by co-treatment with 160 µg/mL of Atofen and NAC (D), DPI (E), PDTC (F), U0126 (G), respectively. Cell adherence was expressed as the fold difference over the untreated control. Values are expressed as mean ± SEM. *P<0.05 compared with baseline. ^#^ *P*<0.05 compared with 160 µg/mL Atofen for 4 h. Abbreviations: DPI, diphenyleneiodonium; HAECs, human aortic endothelial cells; NAC, N-acetylcysteine; PDTC, pyrrolidine dithiocarbamate.

To assess the possible molecular mechanisms of IV iron in CV disease, a second cohort of 60 patients (30 men and 30 women; mean age of 65±12 years) on maintenance HD for more than 6 months was recruited for superoxide production of MNCs, circulating levels of soluble adhesion molecules, and mononuclear-endothelial cell adhesion assays, which served as an index of early atherogenesis *ex vivo* [13]–[16]. Patients who had a malignancy, infectious disease, or sepsis, or hepatobiliary disease were excluded from this study. Initially, all patients were randomly allocated to three groups receiving IV supplementation of Atofen iron or normal saline. IV supplementation with Atofen (40 mg and 80 mg elemental iron diluted in 250 mL of 0.9% saline) or 250 mL of 0.9% saline (control) was administered intravenously for 60 minutes postdialysis once a week for 10 weeks. Blood samples were taken two weeks after administration the last iron dose. All study subjects fasted for 12 hours immediately before the blood samples were taken. Any ongoing medications were continued without changes in dosage during the study. Both the observational and international studies were approved by the Committee on Human Research at Taipei Tzu Chi General Hospital. Written informed consent was obtained from each of the study subjects before study entry.

**Figure 5:**
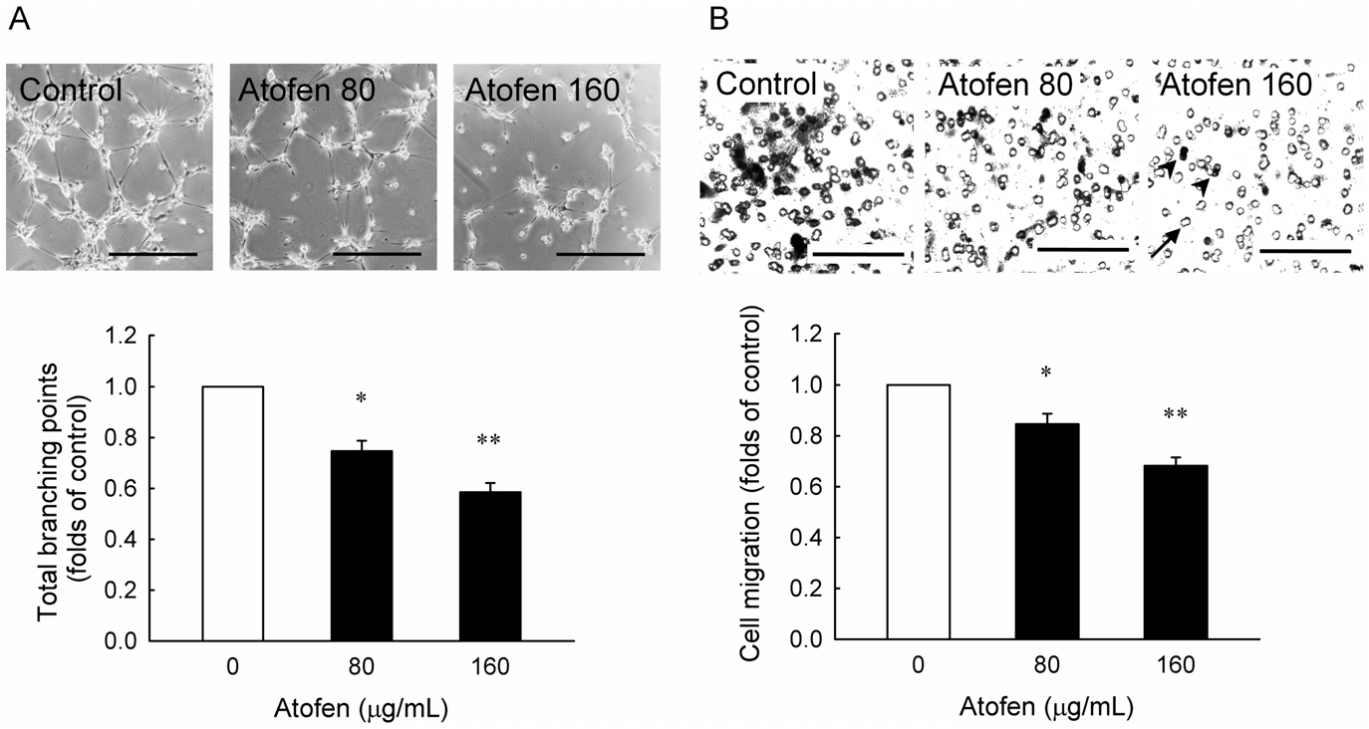
Iron inhibited tube formation and migratory capacity in HAECs. HAECs were treated with ferric chloride hexahydrate (Atofen) for 4 hours in tube formation (A) and 12 hours in cell migration (B). Values are expressed as mean ± SEM. Scale bar represents 50 µm in Figure 5A and 30 µm in Figure 5B. The arrowheads indicate migrated endothelial cells and the arrow indicates membrane pore. *P<0.05, **P<0.001 compared with baseline without Atofen treatment, *n* = 3 in each group. Abbreviations: HAECs, human aortic endothelial cells.

### Isolation of Peripheral Blood Mononuclear Cells

Circulating MNCs from iron-treated HD patients and healthy subjects were isolated and extracted by density ultracentrifugation. In brief, 10 mL peripheral venous blood was drawn into a VACUTAINER® CPT™ Tube with 0.1 M sodium citrate at room temperature at bedside after 30 min of bed rest, with the study subjects in a supine position. The tubes were inverted 10 times gently. The blood was centrifuged and washed with EDTA/Hanks’ balanced salt solution (HBSS) as per the operational instructions [17]. The isolated circulating MNCs were then resuspended with serum-free RPMI 1640 medium for labeling. All samples used in these experiments had at least 95% viability of the isolated MNCs assessed using trypan blue exclusion. The isolation procedures were carried out at room temperature within 2 h of blood collection. After density ultracentrifugation, the separated plasma was frozen to –20°C and stored at that temperature until the analysis of the circulating levels of adhesion molecules.

### Assessment of Superoxide Generated by Mononuclear Cells

Using ultra-weak and luminol-enhanced chemiluminescence, superoxide generation of circulating MNCs was measured [18]. Briefly, after blood sampling, MNCs were immediately isolated by centrifuging (1500×*g*) whole blood in CPT tubes. The MNC suspension was then adjusted to 10^6^ cells/mL (average lymphocytes: monocytes, 93∶7) determined using a Coulter Counter (STKS, Coulter Electronics, Hialeah, FL). After adding 1 mL of 0.25 mm lucigenin to 100 µL of cell suspension, the photon emission at 200–750 nm was measured as the basal photon counts using the BJL-Ultra-Weak Chemiluminescence Analyzer (American Biologics, Chula Vista, CA; sensitivity 1.85×10^–17^ W/cm^2^ count), which were recorded as the basal superoxide generated by MNCs. MNCs (suspended at 1×10^6^/mL) were then activated by adding phorbol-12-myristate-13-acetate molecule (1 µg/mL), and the activated photon counts were recorded for 30 min. The total count of superoxide free radicals was a summation of the basal and the activated counts.

### Measurement of Plasma Levels of Soluble Adhesion Molecules

Plasma levels of human soluble VCAM-1 and ICAM-1 were determined by enzyme-linked immunosorbent assay with commercial kits (BioSource, Camarillo, CA). The procedures were carried out according to the instructions of the manufacturer.

### Cell Culture of Human Aortic Endothelial Cells

Human aortic endothelial cells (Cascade Biologics, Portland, OR) were grown in Medium 200 (Cascade Biologics) supplemented with low-serum growth supplement (Cascade Biologics ) in an atmosphere of 95% air and 5% CO_2_ at 37°C in plastic flasks. At confluence, the cells were subcultured at a 1∶3 ratio and used at passage numbers three through eight in this experiment.

### Mononuclear-endothelial Cell Adhesion Assay

The adhesion assay was performed as previously described [13]–[16], with minor modifications. Before the adhesion assay, human MNCs were labeled with 10 µmol/L of 2′,7′-bis(2-carboxyethyl)-5(6)-carboxyfluorescein acetoxymethyl ester (BCECF-AM; Molecular Probes) in serum-free RPMI 1640 medium for 45 minutes at 37°C in darkness, and the surplus was washed with HBSS. Labeled MNCs (1×10^6^) were then added to each well of control or Atofen-treated HAECs that were confluent in 24-well plates. After incubation at 37°C for 1 hour, non-adherent cells were removed with gentle HBSS washing for 3 times. MNC adhesion to HAECs was counted by an automated fluorometer at an emission of 530 nm and an absorption of 435 nm after cells were lysed with DMSO. The data were expressed as the fold difference over the untreated control in each experiment.

### Western Blot

Confluent HAECs were stimulated with 80 or 160 µg/mL of Atofen with or without N-acetylcysteine (NAC, 10 mmol/L), diphenyleneiodonium (DPI; NADPH oxidase inhibitor, 30 µmol/L), pyrrolidine dithiocarbamate [PDTC; Nuclear factor κB (NF-κB) inhibitor, 100 µmol/L] and U0126 [activator protein-1 (AP-1) inhibitor, 50 µmol/L] for 4 hours. Protein extracts were prepared as previously described [17]. Total protein was quantified by the Bio-Rad protein assay, and processed for Western blot using primary goat antibodies for VCAM-1 and ICAM-1. Afterward, species-directed secondary goat HRP-conjugated monoclonal antibodies were used. Anti-β-actin antibody was used as a loading control. Densitometric analysis using ImageQuant software was conducted to semiquantify the data.

### Tube Formation Assay

Tube formation assay was performed as previously described [19], with minor modifications. Angiogenesis μ-slides (Ibidi Gmbh, Germany) were coated with 10-µl growth factor-containing gel (ECMatrix; Millipore, Billerica, MA) and incubated at 37°C for 1 hour in CO_2_ incubator according to the manufacturer’s instructions. HAECs were plated on the coated plates at 5×10^3^ cells/well in the presence of 0, 80 and 160 µg/ml of Atofen, respectively. After 4 hours of incubation in a 5% CO_2_-humidified atmosphere at 37°C, tube-like formation in the center field was observed by an inverted photomicroscope and captured with a CCD camera. Total branching points were analyzed by WimTube Quantitative Image Analysis (Wimasis, https://mywim.wimasis.com).

### Cell Migration Assay

Cell migration assay was performed as previously described [19], with minor modifications. Assay was conducted using 8.0-µm pore size 24-well Millicell insert (Millipore, Billerica, MA). Nine hundred µl medium with 10% FBS and 0, 80 or 160 µg/ml of Atofen were placed in the lower wells. Cell suspensions (200 µl; 2×10^4^ cells/well) in the presence of different concentrations (0, 80 and 160 µg/ml) of Atofen were added to the upper well, respectively, and cultured at 37°C for 12 hours. Migrated cells on the lower surface of the membrane were fixed in 10% PFA and stained with Hematoxylin. Five random microscopic fields (200X) per well were quantified by ImageJ software.

### Statistical Analysis

All variables are expressed as percentages for categorical data and the mean ± SD (normally distributed data) or medians with interquartile ranges (IQR) (non-normally distributed data). Comparisons between two groups were assessed by Student’s *t* test, Pearson’s chi-square test, or the Mann-Whitney *U* test, as appropriate. Potential differences among the 3 patient groups with different IV iron doses were assessed by multivariate analysis of variance (ANOVA). The Kaplan-Meier method was used to describe survival curves. Cox proportional hazards regression analysis was used to examine the associations of the cumulative dose of IV Atofen with CV events and all-cause mortality. The assumption of proportional hazards was confirmed by a log minus log plot and was met in the Cox models. Univariate and multivariate Cox regression analyses are presented as hazard ratios (HRs) and 95% confidence intervals (CIs). Adjustments for age and gender were initially performed to calculate the adjusted HRs. The multivariate regression analysis was further adjusted for age, sex, the presence of diabetes, hypertension, prior CV disease, hemodialysis duration, serum albumin, hemoglobin and CRP. The *in vitro* results are expressed as the mean ± SEM, and these data were analyzed using one-way ANOVA followed by the LSD test. A *P* value less than 0.05 was considered statistically significant. All statistical analyses were performed using the Statistical Package for the Social Sciences (SPSS), version 16.0 (SPSS Inc., Chicago, IL).

## Results

### IV Atofen Iron Associated with CV Events and All-cause Mortality

Among 1239 HD patients, 332 (27%) had diabetes mellitus and 180 (15%) had a previous history of CV disease. All patients were stratified into two groups with (*n*  =  656) and without (*n*  =  583) supplementation of IV Atofen during 6 months prior to enrollment (Table 1). The patients in the two groups did not differ significantly from each other. The baseline ferritin levels were 566 [IQR: 352, 594] µg/L in non-iron group and 354 [188, 518] µg/L in iron-treated group. The difference between two groups might be due to that the lower the ferritin level, the more the need of IV iron. The follow-up ferritin and hemoglobin levels were 480 [302, 819] µg/L and 10.3±1.8 g/dL at 6 months and 497 [307, 818] µg/L and 10.2±1.7 g/dL at 12 months in non-iron group; and were 392 [254, 583] µg/L and 10.4±1.4 g/dL at 6 months and 400 [228, 583] µg/L and 10.4±1.4 g/dL at 12 months in iron-treated group, respectively. No significant differences in ferritin and hemoglobin levels between the two groups were found during the follow-up.

During a follow-up period of 12 months, 154 patients died, and 83 (53.8%) died of CV-related causes and 71 (46.2%) died of non-CV causes including infection, sepsis, malignancy, gastrointestinal bleeding, chronic obstructive lung disease, and cachexia. There were 207 fatal and non-fatal CV events, i.e. 61 myocardial infarction, 50 stroke, 54 CHF, 15 PVD and 27 sudden death. Figure 1 shows the Kaplan-Meier survival curves for the endpoints of first CV event and the all-cause mortality among the 4 subgroups stratified by the cumulative iron dose within 6 months prior to the study: 0 mg (reference group, *n*  =  583), 40 to 800 mg (Group I, *n*  =  451), 840 to 1600 mg (Group II, *n*  =  111), and 1640 to 2400 mg (Group III, *n*  =  94). The survival analysis showed that higher IV iron doses were significantly associated with a higher risk for CV events (Figure 1A) and a lower probability of survival (Figure 1B) in HD patients.

Cox proportional hazards models (Table 2) corroborated the finding that compared to the reference group, the crude HR was 3.4 (95% CI, 1.9 to 6.0) in Group II and 4.8 (95% CI, 2.9 to 8.4) in Group III for CV events (*P* for trend <0.001), as well as 2.0 (95% CI, 1.1 to 3.4) in Group II and 2.4 (95% CI, 1.3 to 4.1) in Group III for all-cause mortality (*P* for trend  =  0.004). After adjustment for potential confounders, including age, gender, dialysis vintage, comorbidities, and baseline traditional and uremic factors, the adjusted HR was 3.5 (95% CI, 1.9 to 6.1) in Group II and 5.1 (95% CI, 3.0 to 9.9) in Group III for CV events (*P* for trend <0.001), as well as 3.1 (95% CI, 1.6 to 6.3) in Group II and 3.7 (95% CI, 1.8 to 7.7) in Group III for mortality (*P* for trend <0.001). When CV events were confined to the fatal and nonfatal myocardial infarction, stroke and sudden cardiac death, the crude and adjusted HRs showed the similar results (Table 2). In general, a cumulative dose of IV Atofen >800 mg during the 6 months significantly increased the risks for CV events and overall mortality in HD patients.

### Pro-oxidant Effects of IV Atofen Iron *in vivo*

HD patients were randomly assigned the treatment with normal saline (n  =  20), and IV Atofen 400 mg (n  =  20) and 800 mg (n  =  20), respectively. The patients in the three groups did not differ significantly from each other in age, gender, comorbidities, HD vintage, weekly dose of ESA, hemoglobin, serum albumin, and ferritin. The intracellular superoxide production of circulating MNCs was measured in HD patients 2 weeks after a course of IV iron supplementation. As compared to the subjects not receiving IV iron, those receiving IV iron dose of 800 mg significantly had higher total (373±27 *vs*. 582±96×10^3^ count/sec, *P*  =  0.003; Figure 2A) and activated counts of superoxide (353±27 *vs*. 560±95×10^3^ count/sec, *P*  =  0.003; Figure 2B) and the ratio of the activated count to the basal count (18.6±1.9 *vs*. 24.3±2.8, *P*  = 0.049; Figure 2C). Similarly, they had higher plasma levels of soluble VCAM-1 (1081±89 *vs*. 1429±123 ng/mL, *P*  =  0.046; Figure 2D) and ICAM-1 (254±37 *vs*. 439±108 ng/mL, *P*  = 0.028; Figure 2E) than those not receiving IV iron did. Trends of increased superoxide production and plasma adhesion molecules were noted in patients receiving IV iron of 400 mg, but not reach statistical significance. Using the *ex vivo* assay of MNC-endothelial cell adhesion, a significant increase of MNC adhesion to HAECs in a dose-dependent manner was observed in HD patients (1.6±0.2, 4.0±0.4, and 6.1±1.0 folds of U937 control in patients receiving 0 mg, 400 mg and 800 mg, respectively, *P*<0.001; Figure 2F).

### Atofen Iron Upregulated the Expression of VACM-1 and ICAM-1 in HAECs

We examined the *in vitro* effects of Atofen on the expression of endothelial adhesion molecules in HAECs using Western blotting. In the following experiments, the non-cytotoxic Atofen concentrations of ≤160 µg/mL were used based on the MTT cell viability assay (Figure S1) and the report by Zager *et al.* [20]. Atofen at concentrations of 80 and 160 µg/mL significantly upregulated VCAM-1 expression in HAECs at 4 hours (*P*  =  0.045 versus control; Figure 3A). Similarly, Atofen at 160 µg/mL significantly enhanced ICAM-1 expression in HAECs (*P*  =  0.046 versus control; Figure 3B). Co-treatment of HAECs with NAC (10 mmol/L), DPI (30 µmol/L), PDTC (100 µmol/L) and U0126 (50 µmol/L) significantly suppressed the iron-induced expression of VCAM-1 (*P*  =  0.007, 0.006, 0.008 and <0.001 versus Atofen at 160 µg/mL; Figure 3A) and ICAM-1 (*P*  =  0.023, 0.006, 0.041 and 0.004 versus Atofen at 160 µg/mL; Figure 3B), respectively.

### Atofen Iron Enhanced Endothelial Adhesiveness of Circulating MNCs in HAECs

Atofen’s effects in endothelial adhesiveness were further examined, and BCECF-AM–labeled human MNCs and Atofen-treated HAECs were submitted to adhesiveness assays for 4 hours (Figure 4). Atofen induced a dose-dependent increase of endothelial adhesiveness in HAECs at concentrations of 80 to 160 µg/mL (1.48±0.49 and 2.39±0.30 folds of control, *P*  =  0.018 and <0.001; Figure 4). Co-treatment of HAECs with NAC (10 mmol/L), DPI (30 µmol/L), PDTC (100 µmol/L) and U0126 (50 µmol/L) attenuated the iron-induced endothelial adhesiveness (*P*  =  0.003, 0.001, 0.035 and 0.002 versus Atofen at 160 µg/mL; Figure 4), respectively. Accordingly, iron-induced ROS generation via the activation of NAPDH oxidase and the downstream redox-sensitive transcription factors may in part lead to endothelial dysfunction.

### Atofen Iron Inhibited Tube Formation and Migratory Capacity in HAECs

To further examine the Atofen’s effects in angiogenesis and migratory capacity of HAECs, Atofen-treated HAECs were submitted to tube formation assay for 4 hours and Boyden chamber assay for 12 hours, respectively. The quantitative analysis of branching points showed that Atofen induced a dose-dependent decrease of tube formation in HAECs at concentrations of 80 and 160 µg/mL (0.75±0.04 and 0.58±0.04 folds of control, *P*  =  0.011 and <0.001, respectively, Figure 5A). Similarly, Atofen inhibited the migration of HAECs at concentrations of 80 and 160 µg/mL in the Boyden chamber assay (0.85±0.04 and 0.68±0.03 folds of control, *P*  =  0.001 and <0.001, respectively, Figure 5B).

## Discussion

Iron is a cellular transition element, and its ionic forms are prone to participate in one-electron transfer reactions. However, this property also allows iron to generate ROS. In the presence of iron, reactive hydroxyl radicals can be formed via the Fenton reaction and the iron-catalyzed Haber-Weiss reaction [21], [22]. The generated ROS subsequently initiate various types of damage to the human body. In 1981, Sullivan [23] suggested that iron depletion may protect against ischemic heart disease in humans. This “iron hypothesis” has been verified by many epidemiological studies. Bodily iron stores, as measured by the serum ferritin concentration, are positively correlated with the incidence of CV disease [24], [25]. Subsequent cohort studies have found that higher body iron storage may increase the risk for CV disease [26], [27]. In addition, several lines of evidence have indicated an increased iron level in human atherosclerotic plaques [28], [29]. This observation was further supported by Lee *et al*. [30], who found that iron deposition was closely associated with the progression of atherosclerosis in ApoE-deficient mice. Restrictions in dietary iron intake led to a significant reduction of atherosclerotic lesion formation in ApoE-deficient mice. However, the contention that iron participates in vascular inflammation and the subsequent atherosclerosis still remains to be confirmed.

Iron therapy has positive and negative impacts in the clinical outcome of CKD patients. First, anemia can promote left ventricular hypertrophy and augment CV disease in CKD patients. Iron supplementation can improve the efficacy of ESA therapy and increase hemoglobin concentration [3], [4]. Second, over-treatment with iron may increase the risk of CV disease and infection [31], [32]. Third, iron can enhance ROS generation, exaggerate uremia-associated oxidative stress, and subsequently promote atherosclerosis via the oxidation of low-density lipoprotein (LDL) and endothelial dysfunction [33], [34]. Accordingly, the optimal dosage of iron supplementation in CKD patients is still debatable. The main reasons for this are the absence of large clinical, especially randomized controlled, trials, as well as insufficient *in vivo* and *in vitro* experimental evidence.

Atofen is a new iron preparation for parenteral administration to treat anemia and iron deficiency in CKD patients. To our knowledge, this is the first study to assess whether IV Atofen supplementation had detrimental effects on CV events and mortality in HD patients. In this prospective cohort study, compared with those who did not receive IV iron, there were significant dose-dependent risks for fatal and non-fatal CV events and overall mortality among 1239 HD patients. We further observed that supplementation with Atofen of >800 mg over 6 months was significantly associated with poor CV outcome and increased mortality in chronic HD patients. Kalantar-Zadeh *et al*. [9] have observed a J-shaped association between iron dose and outcome, which is different from our observations. Because both were observational studies, the reasons for this discrepancy are mainly due to differences in iron regimens, patient selection, ethnicity, and statistical methodology. In the randomized study, we further found significant increases in ROS production and endothelial adhesion by circulating MNCs from HD patients receiving IV Atofen compared to those without IV iron therapy (Figure 2). Our observations are in line with the finding by Yin *et al.* [13] that the *ex vivo* MNC-endothelial cell adhesion assay can predict the clinical outcome in patients with chronic heart failure.

The molecular mechanisms of the association of iron administration with tissue oxidation or atherosclerosis have not been fully clarified. Zager *et al*. [35] first demonstrated that iron sucrose inhibits aortic endothelial cell proliferation *in vitro*. Carlini *et al*. [36] also found that the inhibitory effect of iron sucrose on endothelial cell proliferation probably results from the overexpression of proteins related to the cell-cycle arrest and apoptosis stress pathways. In rats with a remnant kidney, the IV administration of iron dextran can induce oxidant stress in CV tissues but has no impact on atherosclerotic lesions [37]. Our previous data together with those of others reveal that the activation of NF-κB [14], [38] and AP-1 [39], [40], two major redox-sensitive eukaryotic transcription factors, regulates genes relevant to the expression of adhesion molecules on endothelial cell surface. Furthermore, the activation of these genes can be diminished by various antioxidants [38]–[40]. Intriguingly, our *in vitro* experiments showed that the Atofen-induced expression of adhesion molecules and endothelial adhesiveness were attenuated by the co-treatment of HAECs with NAC and inhibitors of NADPH oxidase, NF-κB, and AP-1 (Figure 3 and 4). Endothelial adhesiveness to MNCs *in vitro* is a reliable cell model for early stages of atherogenesis [14]–[16], [41], indicating that intracellular ROS production by iron therapy may play a central role in atherogenesis. In addition, we also demonstrated that iron can attenuate angiogenesis and inhibit migratory capacity *in vitro*. Taken collectively, the data indicate that IV iron acts as an *in vivo* pro-oxidant to provoke intracellular ROS production, which may induce redox-sensitive transcription pathway activation and adhesion molecule expression, promote the development of atherosclerosis, and even restrain angiogenesis.

Several points from our studies deserve discussion. First, the iron concentrations used (80 and 160 µg/mL) fall within a clinically achievable range of plasma iron concentrations (e.g., ∼150 µg/mL following ∼500 mg IV iron infusion) [20]. Our data provide insight into the molecular mechanisms of iron-related endothelial damage in normal physiologic situations, and an increase in iron levels from a high iron dosage (>800 mg IV infusion) may predispose to more endothelial dysfunction and exacerbate the risk of atherosclerosis in CKD. Second, in the process of MNC-endothelial cell adhesion, arrest and firm adhesion of the MNCs on activated endothelial cell surfaces also depends on the expression of the integrins very late antigen (VLA-4) and lymphocyte function-associated antigen-1 (LFA-1). Kartikasari *et al.* [42] found that VLA-4 and LFA-1 were upregulated on monocytes after 1 hour of 10 µmol/L Fe(III) citrate treatment, suggesting that iron may also enhance monocyte activation. Third, NADPH oxidase-derived ROS, in particular, superoxide radicals and their dismutation product, hydrogen peroxide, have been implicated in the development of human atherosclerotic lesions [43]. Clinically, there is a close relationship between cellular iron status and NADPH oxidase activity. Kurtoglu *et al.* [44] reported that the activity of NADPH oxidase was significantly lower in patients with iron-deficient anemia, and iron supplementation increased NADPH oxidase activity. Li *et al.* [45] found that iron increased endothelial NADPH oxidase activity by increasing p22^phox^ gene transcription. These results support the findings in our study. We demonstrated that IV iron can significantly enhance the total and activated superoxide production of circulating MNCs isolated from HD patients. Our *in vitro* study further demonstrated that the co-treatment of iron-stimulated HAECs with an NADPH oxidase inhibitor ameliorated the iron-enhanced expression of adhesion molecules and endothelial adhesiveness. It implies that antioxidants may potentially protect against iron-mediated endothelial dysfunction in clinical practice.

Some limitations of this study should be acknowledged. There was no random allocation of treatment in our study on CV outcome and mortality. The nature of the observational cohort study might bear the possibility of selection bias. Nevertheless, the two groups of patients, those receiving and not receiving IV iron, did not differ significantly from each other in the baseline demographic and laboratory data. Furthermore, in analyzing the association of iron dosage with the end points, we used multivariate Cox hazards models to adjust for the likelihood of potential confounders. Unlike the previous studies [7]–[9], the single IV iron regimens applied in our cohort reduced the potential discrepancy that can result from the use of various IV iron medications and minimized the confounders in some associations. Finally, the type of vascular access, native arteriovenous fistula, arteriovenous graft or temporary catheter might represent an additional confounding variable. Despite the above-mentioned limitations, our data from the randomized controlled study and *in vitro* experiments still provide a causal interrelation among iron supplementation, ROS production and endothelial dysfunction, and provided the pathomechanism of IV iron-induced artherogenesis in HD patients.

In summary, our prospective cohort study demonstrates that high cumulative IV iron supplementation significantly increased the risks for fatal and non-fatal CV events and all-cause mortality in chronic HD patients, especially in those receiving Atofen >800 mg over 6 months. In addition, IV-administered iron acts as a pro-oxidant *in vivo* to enhance superoxide production of circulating MNCs, soluble adhesion molecule levels in the plasma, and the endothelial adhesion of circulating MNCs in chronic HD patients. Furthermore, our *in vitro* experiments showed that Atofen increased adhesion molecule expression and aggravated MNC-endothelial cell adhesion in cultured HAECs. Our findings may indicate a novel role of Atofen iron in exacerbating atherosclerosis in HD patients. From a clinical point of view, to confront the enhanced oxidant stress and subsequent vascular injury caused by IV-administered Atofen, long-term, randomized controlled studies of IV Atofen are needed to better define the clinically safe dose, if any, of this therapy.

## Supporting Information

Figure S1 **Cell viability of human aortic endothelial cells 4 h following culture with various concentrations of ferric chloride hexahydrate (Atofen), as determined by the MTT assay.** The percentage of cell viability in the Atofen-treated groups was compared with the untreated group (cell viability  = 100%). The data are expressed as the mean ± SEM from three independent experiments.
(PDF)
