# Correction: Intravenous Ferric Chloride Hexahydrate Supplementation Induced Endothelial Dysfunction and Increased Cardiovascular Risk among Hemodialysis Patients

**DOI:** 10.1371/annotation/828aa889-2cb2-4bff-91d3-e247d7da2e14

**Published:** 2013-05-14

**Authors:** Ko-Lin Kuo, Szu-Chun Hung, Yao-Ping Lin, Ching-Fang Tang, Tzong-Shyuan Lee, Chih-Pei Lin, Der-Cherng Tarng

The Table 1 that was presented is incorrect. The correct version is available below.

Table 1: http://plosone.org/corrections/pone.0050295.t001.cn.tif

