# Supplementary material for: Intravenous Ferric Chloride Hexahydrate Supplementation Induced Endothelial Dysfunction and Increased Cardiovascular Risk among Hemodialysis Patients
Source: PLoS One. 2012 Dec 5;7(12):e50295. doi: 10.1371/journal.pone.0050295 (PMC3515606; doi:10.1371/journal.pone.0050295)
Supplement: Figure S1 — Cell viability of human aortic endothelial cells 4 h following culture with various concentrations of ferric chloride hexahydrate (Atofen), as determined by the MTT assay. The percentage of cell viability in the Atofen-treated groups was compared with the untreated group (cell viability = 100%). The data are expressed as the mean ± SEM from three independent experiments. (PDF) [file pone.0050295.s001.pdf]

Supplementary Data

Methods

MTT assay for cell viability

The 3-(4,5-dimethylthiazol-2-yl)-2,5-diphenyl tetrazolium bromide (MTT) assay (Sigma Chemical Co., St. Louis, MO, USA) was used to measure cell viability. The principle of this assay is that mitochondrial dehydrogenase in viable cells reduces MTT to a blue formazan. Briefly, cells were grown in 96-well plates and incubated with various concentrations of ferric chloride hexahydrate (Atofen®; Uji Pharmaceutical Co. Ltd., Japan) for 4 h. After washing human aortic endothelial cells (HAECs) with phosphate-buffered saline (PBS), 100 μL of medium containing MTT (0.5 mg/mL) was added to each well for incubation at 37°C for 4 h. The medium was then carefully removed to avoid disturbing the formazan crystals that had formed. One hundred microliters of DMSO, which can solubilize the formazan crystals, was added to each well, and the absorbance of the solubilized blue formazan was read at 540 nm using a microplate reader (Multiskan Ex, Thermo Labsystems, Beverly, MA). DMSO was the blank. The reduction in optical density caused by the drug was used as a measurement of cell viability. Optical density in each Atofen-treated group was normalized to optical density of the cells incubated in the control medium, which were considered 100% viable (the untreated group).

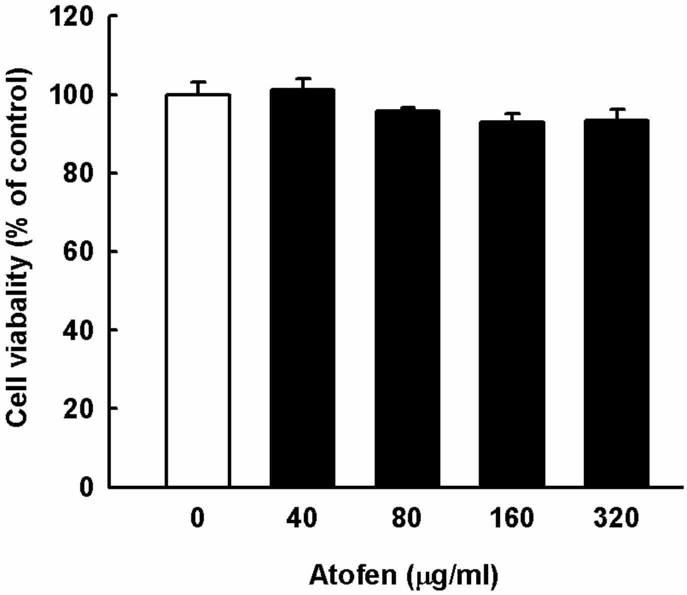

Figure S1. Cell viability of human aortic endothelial cells 4 h following culture with various concentrations of ferric chloride hexahydrate (Atofen), as determined by the MTT assay. The percentage of cell viability in the Atofen-treated groups was compared with the untreated group (cell viability = 100%). The data are expressed as the mean ± SEM from three independent experiments.
